# Neighborhood Socioeconomic Status and Use of Colonoscopy in an Insured Population – A Retrospective Cohort Study

**DOI:** 10.1371/journal.pone.0036392

**Published:** 2012-05-02

**Authors:** Chyke A. Doubeni, Guruprasad D. Jambaulikar, Hassan Fouayzi, Scott B. Robinson, Margaret J. Gunter, Terry S. Field, Douglas W. Roblin, Robert H. Fletcher

**Affiliations:** 1 Department of Family Medicine and Community Health, Medical School, University of Massachusetts, Worcester, Massachusetts, United States of America; 2 Meyers Primary Care Institute/Reliant Medical Group/Fallon Community Health Plan, Department of Medicine, Medical School, University of Massachusetts, Worcester, Massachusetts, United States of America; 3 Premier Inc., Charlotte, North Carolina, United States of America; 4 LCF Research, Albuquerque, New Mexico, United States of America; 5 The Center for Health Research-Southeast, Kaiser Permanente Georgia, Atlanta, Georgia, United States of America; 6 Department of Population Medicine, Harvard Medical School, Boston, Massachusetts, United States of America; The University of Texas M. D. Anderson Cancer Center, United States of America

## Abstract

**Background:**

Low-socioeconomic status (SES) is associated with a higher colorectal cancer (CRC) incidence and mortality. Screening with colonoscopy, the most commonly used test in the US, has been shown to reduce the risk of death from CRC. This study examined if, among insured persons receiving care in integrated healthcare delivery systems, differences exist in colonoscopy use according to neighborhood SES.

**Methods:**

We assembled a retrospective cohort of 100,566 men and women, 50–74 years old, who had been enrolled in one of three US health plans for ≥1 year on January 1, 2000. Subjects were followed until the date of first colonoscopy, date of disenrollment from the health plan, or December 31, 2007, whichever occurred first. We obtained data on colonoscopy use from administrative records. We defined screening colonoscopy as an examination that was not preceded by gastrointestinal conditions in the prior 6-month period. Neighborhood SES was measured using the percentage of households in each subject's census-tract with an income below 1999 federal poverty levels based on 2000 US census data. Analyses, adjusted for demographics and comorbidity index, were performed using Weibull regression models.

**Results:**

The average age of the cohort was 60 years and 52.7% were female. During 449,738 person-years of follow-up, fewer subjects in the lowest SES quartile (Q1) compared to the highest quartile (Q4) had any colonoscopy (26.7% vs. 37.1%) or a screening colonoscopy (7.6% vs. 13.3%). In regression analyses, compared to Q4, subjects in Q1 were 16% (adjusted HR = 0.84, 95% CI: 0.80–0.88) less likely to undergo any colonoscopy and 30%(adjusted HR = 0.70, CI: 0.65–0.75) less likely to undergo a screening colonoscopy.

**Conclusion:**

People in lower-SES neighborhoods are less likely to undergo a colonoscopy, even among insured subjects receiving care in integrated healthcare systems. Removing health insurance barriers alone is unlikely to eliminate disparities in colonoscopy use.

## Introduction

In the United States, people in low socioeconomic status are more likely than those from higher socioeconomic groups to be diagnosed with, and die from, colorectal cancer [1]–[5]. Screening with tests such as colonoscopy that are recommended by national groups in the United States has been shown to reduce the risk of incidence of or death from colorectal cancer [6]–[11]. People from low socioeconomic groups are less likely to undergo colorectal cancer screening [12]–[17]. This suggests that socioeconomic disparities in death from colorectal cancer may result, in part, from unequal access to and/or unequal use of screening. Consequently, some public health programs aimed at eliminating disparities in death from colorectal cancer have focused on providing greater access to screening [18].

Although observational studies and clinical trials on the efficacy of colonoscopy are ongoing [19], direct and indirect evidence including studies in high risk populations support its current use [7], [8], [20]. Colonoscopy affords the endoscopist direct visualization of the entire colon from the rectum to the cecum while making it possible to remove most precancerous lesions and some early cancers found at the time of screening [21]. Colonoscopy is also the recommended diagnostic test in patients with positive findings on other screening tests [22]. As a result of these appealing properties, some groups believe and promote colonoscopy as the best screening test for colorectal cancer [21]. Colonoscopy is now the most commonly used colorectal cancer screening test in the United States [12]–[14], and its use is also increasing in other countries [23].

Colonoscopy is also the most expensive and invasive of the tests currently recommended by the US Preventive Services Task Force [24]. It is not surprising therefore that population-based studies have consistently shown that people in low socioeconomic status are less likely to undergo colonoscopy when compared to those from higher socioeconomic groups [14]–[17]. The socioeconomic disparity in colonoscopy use is believed to be due, in part, to differences in health insurance coverage or access to health care services. That belief is supported by studies showing a consistently strong association between the type of health insurance coverage and colonoscopy use, even among people in Medicare for whom screening colonoscopy has been a covered benefit since 2001 [14], [17], [25]. Studies have also shown that differences in colonoscopy use by socioeconomic status, at the individual or area level, persist even after accounting for type of health insurance coverage [14], [26].

Those findings suggest that providing health insurance coverage and removing out-of-pocket cost for colonoscopy, as mandated by the Affordable Care Act [12], [27], may not eliminate socioeconomic disparities in use of colonoscopy in the United States. Colonoscopy is a complex screening test involving extensive bowel preparation. Thus, other financial and non-financial barriers to health care for low-income populations including cultural barriers, the need for time off work or other competing social needs, and difficulties in navigating healthcare systems [28], may continue to limit colonoscopy use for low-income populations. However, it remains largely unknown whether socioeconomic differences in colonoscopy use persist when health insurance and out-of-pocket cost barriers are removed.

Integrated healthcare delivery systems provide an appropriate setting to evaluate the potential impact of removing out-of-pocket cost barriers on socioeconomic disparities in colonoscopy use. In this study, we evaluated whether the use of colonoscopy in populations with similar health insurance coverage with little or no out-of-pocket cost and an available usual place of health care differed according to neighborhood socioeconomic status. Our study population, unlike Medicare, was comprised mostly of employed persons who were insured and received care from a common healthcare provider network.

## Methods

### Ethics Statement

This study was reviewed by the Institutional Review Boards of the University of Massachusetts Medical School (Worcester, MA) and the participating sites. Because the study did not involve contact with study subjects, it was considered exempt from a full human subjects review and from obtaining informed consent.

### Study Design and Population

This was a cohort study that used electronic administrative and clinical data for persons who were, on January 1, 2000, members of Reliant Medical Group/Fallon Community Health Plan in Massachusetts, Kaiser Permanente Georgia, or Lovelace Health System in New Mexico. These health care systems are part of the HMO Cancer Research Network, which currently has fourteen member organizations that use varying models of health care delivery. The health plans have programs to promote preventive healthcare, including periodic reminders on cancer screening. All subjects were insured and had access to care from a common clinical provider network at each study site. Colorectal cancer screening at each site was provided according to prevailing national recommendations as a covered benefit to the members.

The historical cohort for this study was comprised of men and women who were between the ages of 50 and 74 years, and had been members of one of three participating health plans for at least 1 calendar year (1999) as of January 1, 2000. We excluded all subjects who had a diagnosis of colorectal cancer using a broad definition of one record, or more, of any International Classification of Disease, 9^th^ Edition, Clinical Modification (ICD9) code for colorectal cancer in the 1999 calendar year period in the clinical databases of the health plan. We then tracked the utilization history of the subjects until they received their first colonoscopy, the last date of known enrollment in the health plan, or the end of the study period (December 31, 2007), whichever occurred first.

### Data on Use of Colonoscopy

Data, including dates, on medical diagnoses and procedures received by subjects were obtained from the electronic administrative and clinical databases using ICD9, Common Procedure Terminology (CPT) and Healthcare Common Procedure Coding System (HCPCS) codes. The use of colonoscopy was identified using the following codes: ICD9 – 45.23, 45.25, 45.42, 45.43; CPT – 44388–9, 44392–4, 45378, 45380, 45382, 45383–45385, 45388; and HCPCS – G0105 and G0121. We also created a variable for screening colonoscopy use, which was defined as a colonoscopy that was not preceded by a gastrointestinal condition in the prior 6 months, using an approach like that described by Ko et al. [29]. The presence of gastrointestinal conditions was based on a record of any of the following ICD9 codes in electronic data: gastrointestinal bleeding (456.0, 456.20, 530.82, 531.xx-534.xx, 535.01–535.41, 535.51, 535.61, 562.02, 562.03, 562.12, 562.13, 578.0, 578.1, or 578.9), occult bleeding (792.1), anemia (280 or 285.9), abdominal pain (787.3, 789.0 or 789.6), weight loss (783.2), inflammatory bowel disease (555.xx-556.xx, 558.2 or 558.9), or history of colon polyps (V12.72, 211.3, or 211.4).

### Data on Neighborhood Socioeconomic Status

We obtained data on socioeconomic factors on each subject through linkage of residential addresses to 2000 US decennial census data at the census-tract level of aggregation. For this study, we used the percentage of households in the census tract with incomes below the 1999 federal poverty level as our measure of neighborhood socioeconomic status. This measure was highly correlated (r = 0.97, p-value<0.001) with a summary index of neighborhood socioeconomic deprivation derived by performing principal factor analyses on 19 census variables as described in previous publications [30], [31]. The cohort was divided into quartiles based on household poverty levels, such that subjects in census-tracts with the highest levels of household poverty (or lowest socioeconomic status) in the study comprised quartile 1 and census-tracts in the highest socioeconomic group comprised quartile 4.

### Data on Covariates

We also obtained electronic data on subjects' age, sex, race (whites, blacks, others, or missing), and length of health plan enrollment. The Deyo modification of the Charlson comorbidity index (categorized as 0, 1, and 2+) was calculated using the data on medical diagnoses and procedures during the 1999–2000 calendar years period [32].

### Statistical Analysis

We compared the characteristics of the cohort according to quartiles of neighborhood socioeconomic status using the Chi-square test for categorical variables and analysis of variance test for continuous variables. We used parametric survival models with Weibull distribution and gamma frailties to estimate the association between quartiles of neighborhood socioeconomic status (census-tract household poverty level) and use of any colonoscopy or screening colonoscopy during the 8-year follow-up period. Frailty models allowed us to evaluate geographic variability in colonoscopy use across census-tracts with the likelihood ratio test. We also accounted for clustering of subjects within census-tracts in the analyses. Multivariate analyses adjusted for age at baseline, sex, modified Charlson comorbidity index, study site (health plan), and length of health plan enrollment. We also conducted analyses stratified by health plan and age (50–64 vs. 65–74 years). The age cutoff for the stratified analyses was based on the Medicare age eligibility criterion. About 50% of the subjects in the fourth quartile and 73% in the first quartile had missing data on race. Therefore, we did not include race or ethnicity in our estimation models. All analyses were performed using STATA version 12.1 (StataCorp 2011. College Station, TX: StataCorp LP).

## Results

There was a total of 100,566 subjects from the three health plans in 1,536 census-tracts analyzed for this study, of whom 53,042 (52.7%) were female, the average age was 60 (range 50–74) years and 69,286 (68.9%) were younger than 65 years of age. The mean follow-up on the study was 4.5 years. **Table 1** shows the characteristics of cohort by quartiles of neighborhood socioeconomic status (SES). Subjects in low-SES (higher poverty) areas were older and more likely to be female. The length of enrollment in the health plans also varied across SES quartiles: 9,906 (39.7%) subjects in the first (lowest SES) quartile remained enrolled in the health plan for the full 8 years of follow-up on the study compared to 12,562 (48.4%) in the fourth (highest SES) quartile.

**Table 1:** Characteristics of the study cohort according to neighborhood socioeconomic status, N = 100,566.

	Quartiles of neighborhood socioeconomic status*			
*Characteristics, n (%)*	Quartile 1 N = 24,959	Quartile 2 N = 24,503	Quartile 3 N = 25,148	Quartile 4 N = 25,956
**Age, years**				
50–54	7523 (30.1)	7353 (30.0)	7804 (31.0)	8735 (33.7)
55–59	5364 (21.5)	5234 (21.4)	5289 (21.0)	5875 (22.6)
60–64	4170 (16.7)	3956 (16.1)	3937 (15.7)	4046 (15.6)
65–69	4188 (16.8)	4249 (17.3)	4418 (17.6)	4056 (15.6)
70–74	3714 (14.9)	3711 (15.1)	3700 (14.7)	3244 (12.5)
**Female**	13503 (54.1)	13054 (53.3)	13262 (52.7)	13223 (50.9)
**Study site/Health plan**				
A	5150 (20.6)	8298 (33.9)	12031 (47.8)	9904 (38.2)
B	4652 (18.6)	5887 (24.0)	7183 (28.6)	9567 (36.9)
C	15157 (60.7)	10318 (42.1)	5934 (23.6)	6485 (25.0)
**Enrollment history**				
Percent enrolled at year 5	12844 (51.5)	14503 (59.2)	16562 (65.9)	16707 (64.4)
Percent enrolled at year 8	9906 (39.7)	11090 (45.3)	12701 (50.5)	12562 (48.4)
**Modified Charlson comorbidity Index at baseline**				
0	21435 (85.9)	20097 (82.0)	19489 (77.5)	21433 (82.6)
1	1801 (7.2)	2249 (9.2)	2949 (11.7)	2410 (9.3)
2+	1723 (6.9)	2157 (8.8)	2710 (10.8)	2113 (8.1)

Note: The p-value statistic for heterogeneity across categories was <0.001 on all variables.

* Neighborhood socioeconomic status was measured by the percentage of households in the census-tract level below the 1999 federal poverty levels based on 2000 US census. Quartile 1 corresponds to the lowest socioeconomic (highest household poverty rates) group and Quartile 4 corresponds to the census tracts with the highest socioeconomic status relative to others in the study.

During 449,738 person-years of follow-up on the study, 33,630 (33.4%) subjects had at least 1 colonoscopy including 11,093 (11.0%) screening colonoscopies. **Table 2** compares the use of any colonoscopy by neighborhood SES. Compared to the fourth quartile (n = 9,617; 37.1%), fewer subjects in the second (n = 7,859; 32.1%) or first (n = 6,658; 26.7%) SES quartile had any colonoscopy. **Table 2** also shows the results of Weibull regression models analyses on the association between neighborhood SES and any colonoscopy use. Compared to subjects in the fourth quartile, subjects' likelihood of colonoscopy use decreased with decreasing neighborhood SES. In the unadjusted model, those in the first quartile were about 19% (hazard ratio [HR] = 0.81, 95% confidence interval [CI]: 0.77–0.86) less likely to have had any colonoscopy than subjects in the fourth quartile. The association between SES and colonoscopy use was stable (HR = 0.84, 95% CI: 0.80–0.88) to adjustment for age, gender, study site, modified Charlson comorbidity index, and number of years of enrollment. The use of colonoscopy also varied across the census-tracts in the study (likelihood ratio test p-value<0.001).

**Table 2:** Association between neighborhood socioeconomic status and use of any colonoscopy, 2000–2007.

Quartiles of neighborhood socioeconomic status*	Colonoscopies, n (%)	Hazard ratio (95% confidence interval)	
		Unadjusted	Adjusted†
1st quartile	6658 (26.7)	0.81 (0.77–0.86)	0.84 (0.80–0.88)
2nd quartile	7859 (32.1)	0.89 (0.84–0.94)	0.89 (0.86–0.93)
3rd quartile	9496 (37.8)	0.99 (0.94–1.04)	0.97 (0.93–1.01)
4th quartile	9617 (37.1)	1.00	1.00

* Neighborhood socioeconomic status was measured by the percentage of households in the census-tract level below the 1999 federal poverty levels based on 2000 US census. Quartile 1 corresponds to the lowest socioeconomic (highest household poverty rates) group and Quartile 4 corresponds to the census tracts with the highest socioeconomic status relative to others in the study.

† Estimates were adjusted for age at baseline, gender, modified Charlson comorbidity index at baseline, number of years of enrollment and health plan. Likelihood-ratio test p-value for heterogeneity across census tract was <0.001.

**Tables 3** compares the use of screening colonoscopy across SES quartiles. Fewer subjects in the first quartile (n = 1,908; 7.6%) or second (n = 2,463; 10.1%) than in the fourth quartile (n = 3,447; 13.3%) had a screening colonoscopy during the follow-up period. In unadjusted Weibull regression analyses, subjects in the first quartile were about 34% (HR = 0.66, 95% CI: 0.61–0.72) less likely to have had a screening colonoscopy compared to those in the fourth quartile. In the adjusted analyses, compared to the fourth quartile, subjects in the first quartile were about 30% (HR = 0.70, 95% CI: 0.65–0.75) less likely to have had a screening exam. Similar to analysis on any colonoscopy, there was statistically significant heterogeneity in the use of screening colonoscopy across the census-tracts (likelihood ratio test p-value<0.001).

**Table 3:** Association between neighborhood poverty and use of screening colonoscopy, 2000–2007.

Quartiles of neighborhood socioeconomic status*	Colonoscopies, n (%)	Hazard ratio (95% confidence interval)	
		Unadjusted	Adjusted†
1st quartile	1908 (7.6)	0.66 (0.61–0.72)	0.70 (0.65–0.75)
2nd quartile	2463 (10.1)	0.79 (0.73–0.86)	0.80 (0.75–0.85)
3rd quartile	3275 (13.0)	0.94 (0.87–1.01)	0.92 (0.86–0.97)
4th quartile	3447 (13.3)	1.00	1.00

* Neighborhood socioeconomic status was measured by the percentage of households in the census-tract level below the 1999 federal poverty levels based on 2000 US census. Quartile 1 corresponds to the lowest socioeconomic (highest household poverty rates) group and Quartile 4 corresponds to the census tracts with the highest socioeconomic status relative to others in the study.

† Estimates were adjusted for age at baseline, gender, modified Charlson comorbidity index at baseline, number of years of enrollment and health plan. Likelihood-ratio test p-value for heterogeneity across census tract was <0.001.

**Table 4** shows analyses on the association between neighborhood SES and colonoscopy use stratified by age and study sites (health plan). In the adjusted analyses among subjects 50–64 years of age (n = 69,286), compared to the fourth quartile, subjects in the first quartile were about 17% (HR = 0.83, 95% CI: 0.78–0.87) less likely to have any colonoscopy and 30% (HR = 0.70, 95% CI: 0.65–0.76) less likely to have had a screening exam. The findings were similar from analysis on subjects 65–74 years of age (n = 31,280) for any colonoscopy (HR = 0.88, 95% CI: 0.83–0.93) or screening colonoscopy (HR = 0.71, 95% CI: 0.63–0.80). We also found similar results across the 3 health plans: neighborhood SES was significantly associated with colonoscopy use in a dose response fashion irrespective of the health plan analyzed.

**Table 4:** Association between neighborhood socioeconomic status and use of colonoscopy according to age and health plan, 2000–2007.

	Adjusted hazard ratios (95% confidence interval)†				
Colonoscopy outcome and quartiles of neighborhood socioeconomic status*	According to age		According to health plan		
	50–64 years	65–74 years	A	B	C
**Any colonoscopy**	N = 69,286	N = 31,280	N = 35,383	N = 27,289	N = 37,894
1st quartile	0.83 (0.78–0.87)	0.88 (0.83–0.93)	0.82 (0.77–0.87)	0.83 (0.77–0.90)	0.84 (0.77–0.91)
2nd quartile	0.89 (0.84–0.93)	0.92 (0.87–0.98)	0.92 (0.87–0.98)	0.87 (0.81–0.94)	0.90 (0.82–0.99)
3rd quartile	0.95 (0.90–0.99)	1.01 (0.96–1.07)	1.00 (0.95–1.05)	0.94 (0.87–1.01)	0.96 (0.86–1.06)
4th quartile	1.00	1.00	1.00	1.00	1.00
**Screening colonoscopy**					
1st quartile	0.70 (0.65–0.76)	0.71 (0.63–0.80)	0.70 (0.63–0.78)	0.68 (0.59–0.77)	0.71 (0.63–0.81)
2nd quartile	0.80 (0.75–0.87)	0.81 (0.72–0.90)	0.85 (0.77–0.93)	0.74 (0.66–0.84)	0.82 (0.72–0.94)
3rd quartile	0.93 (0.87–0.99)	0.90 (0.81–1.00)	0.99 (0.91–1.07)	0.84 (0.75–0.93)	0.93 (0.81–1.08)
4th quartile	1.00	1.00	1.00	1.00	1.00

* Neighborhood socioeconomic status was measured by the percentage of households in the census-tract level below the 1999 federal poverty levels based on 2000 US census. Quartile 1 corresponds to the lowest socioeconomic (highest household poverty rates) group and Quartile 4 corresponds to the census tracts with the highest socioeconomic status relative to others in the study.

† Estimates were adjusted for age at baseline, gender, modified Charlson comorbidity index at baseline, number of years of enrollment and health plan. Likelihood-ratio test p-value for heterogeneity across census tract was <0.001.

## Discussion

In this study, we examined the association between neighborhood socioeconomic status (defined by percent of households below the federal poverty level) and use of colonoscopy in an insured population. A unique characteristic of the population was that, within each study site, subjects were in the same health plan and served by the same clinical provider network, and thus the ability to acess a usual place of care. The health systems included in this study provide colonoscopy as a covered benefit to members and also had systems to encourage members to use preventive health services. These characteristics of the healthcare environment would be expected to mitigate barriers to the use of colonoscopy for persons in low-socioeconomic status and thus lessen disparities.

We found significant socioeconomic differences in the use of colonoscopy. Persons residing in the lowest SES neighborhoods were 16% less likely to undergo any colonoscopy relative to those in the highest SES neighborhoods. This association was even stronger (30%) when only screening colonoscopy was considered. Socioeconomic differences in colonoscopy use observed in the general population could be attributed to the fact that people receive care from diverse clinical provider networks and have differing types of health insurance coverage [12]–[17]. However, our results suggest that simply having health insurance and a usual place of care are not enough to eliminate socioeconomic disparities in the use of colonoscopy use, and that other factors related to poverty limit or restrict colonoscopy use. Elimination of disparities concerning colorectal cancer for socioeconomically disadvantaged populations will require measures that also address other economic, social and cultural barriers to receipt of health care services.

Our findings, together with existing research, suggest the need for effective patient navigation or outreach programs in integrated healthcare delivery systems to address disparities in colonoscopy use. Also, performance incentives based on Healthcare Effectiveness Data and Information Set (HEDIS) measures, as practiced in some health care systems, could be effective means to address the disparities we found [33]. However, the effectiveness of such programs in eliminating socioeconomic disparities in colonoscopy use among people receiving their health care under the auspices of healthcare delivery systems is unproven and needs to be studied.

There are few published studies on area-level variations in the use of colonoscopy; very few, if any, of such studies have been conducted within integrated healthcare delivery system settings. Our findings, however, are consistent with studies on Medicare populations showing disparities based on individual-level measures of socioeconomic status [14], [17]. Klabunde et al., using 2008 US National Health Interview Survey data, found that about 34% of screening-eligible adults in families at ≤200% of federal poverty level had colonoscopy compared to 58% among those at ≥500% of federal poverty level, a 1.7-fold difference [13]. A study using Missouri Behavioral Risk Factor Surveillance Survey data also reported that the use of colorectal cancer screening varied across zip-code areas as well as by zip-code poverty levels; socioeconomic differences remained even after adjustment for health insurance type and having a primary care provider [26]. Our study examined socioeconomic differences in colonoscopy use in a smaller area of aggregation and found both geographic and socioeconomic variations.

The population we studied was comprised predominantly of employed persons from varying socioeconomic backgrounds. Socioeconomic status is a predictor of both where people live and colorectal cancer testing. Therefore, the findings from this study are a reflection of the socioeconomic diversity among the members of the respective health care systems, which might influence screening colonoscopy use in several possible ways in integrated healthcare delivery systems. People from low-socioeconomic groups may be late adopters [34] of colonoscopy which has been used increasingly for routine screening in recent years, and they may have greater ambivalence about the balance of risks and benefits associated with the test. Factors related to poverty such as resource deprivation as posited in the Deprivation-Amplification hypothesis [35], cultural barriers, and difficulty navigating healthcare systems, may act separately or together to restrict access to colonoscopy [28]. The need for transportation to and from the procedure may disproportionately impact those from lower socioeconomic groups, despite having health insurance. People in low socioeconomic status may also experience a greater negative impact from the time and preparation required for colonoscopy and the burden of taking time off from work.

Although the enrollees were insured, the health plans provide a variety of insurance programs with varying levels of coverage for colonoscopy. The burden of out-of-pocket expenses may disproportionally impact low-income people in the health plans studied. The co-pay for colonoscopy in the health plans across all coverage types during the study period was between $0 and $200. A study of 106 health plans in the United States found that out-of-pocket costs of $300 or greater negatively affect colonoscopy use [36]. This suggests that potential differences in co-pay among study subjects are unlikely to explain our findings. Further, analyses stratified on health plan or age confirmed the results.

There may also be socioeconomic differences in the patient-physician communication [37] around colorectal cancer screening, including differences in physician recommendation for colonoscopy, that may account for some of the differences observed. It is also possible that people of a lower socioeconomic status may experience higher levels of mistrust of the medical care system and may have greater difficulties gaining access to health care systems despite having health insurance [38]. Other barriers may include embarrassment, lack of knowledge and cultural factors [39]–[41]. Analyses of these potential barriers for insured populations are beyond the scope of our study, but warrant further investigation.

Although we were not able to determine if our findings were solely the result of patient, provider or healthcare system factors, the findings do suggest the need to pay greater attention to the preventive care needs of all people who reside in socioeconomically deprived neighborhoods regardless of whether or not they have health insurance. Area-based socioeconomic measures are readily accessible and can be utilized to guide the implementation of patient navigator programs and reminder systems [42]–[44].

Our study has other limitations. We relied on codes in electronic administrative and clinical databases to ascertain colonoscopy utilization and did not have precise measurements of screening colonoscopy. This might have led to a non-differential misclassification of the outcome, thus attenuating differences. A more accurate measurement may have found larger socioeconomic disparities in colonoscopy use.

We did not have individual-level measures of socioeconomic status determinants such as education, income or occupation. Therefore, the observed neighborhood effects cannot be interpreted as being independent of individual-level socioeconomic factors. However, given the challenges of collecting information on individual-level socioeconomic data, our findings reinforce the value of area-level socioeconomic data as being a suitable approach for assessing socioeconomic disparities in colorectal cancer screening. Further, while neighborhood poverty level may not fully reflect the poverty level of individuals within an area and its effect on their use of colonoscopy, the contextual factors captured by neighborhood measures provide information beyond characteristics of individuals alone. Prior research also shows that neighborhood socioeconomic measures have similar predictive power as individual measures [2]. Another limitation was that over one-half of the subjects had missing data on race. We were therefore unable to account for potential confounding by race on the associations studied. However, prior research suggests that inclusion of race in the analyses would not substantially alter our results [14], [17].

Finally, we did not follow subjects for 10 years, which is the recommended interval for screening colonoscopy. This may have resulted in an underestimation of the true socioeconomic effect if subjects from high-SES neighborhoods had continued to have higher rates of colonoscopy use past the 8 years of follow-up on this study.

In conclusion, this study found that, among insured persons receiving care in integrated health care delivery systems, those residing in poor neighborhoods were less likely to have had a colonoscopy compared to persons in high-SES neighborhoods, despite receiving care from a common clinical provider network. Therefore, providing health insurance or even free colonoscopy services, a sound public health policy, may not eliminate socioeconomic disparities in colonoscopy use without attention to other barriers. Future studies of financial and non-financial barriers to colonoscopy use are needed to identify effective approaches to eliminate disparities in colonoscopy use in insured populations.
